# Horizontal Bone Augmentation with Natural Collagen Porcine Pericardium Membranes: A Prospective Cohort Study

**DOI:** 10.3390/medicina61101814

**Published:** 2025-10-10

**Authors:** Pier Paolo Poli, Luca Giboli, Mattia Manfredini, Shahnavaz Khijmatgar, Francisley Ávila Souza, Carlo Maiorana

**Affiliations:** 1 Department of Biomedical, Surgical and Dental Sciences, University of Milan, 20122 Milan, Italy; luca.giboli@unimi.it (L.G.); mattia.manfredini@unimi.it (M.M.); carlo.maiorana@unimi.it (C.M.); 2 Implant Center for Edentulism and Jawbone Atrophies, Maxillofacial Surgery and Dental Unit, Fondazione IRCCS Cà Granda Ospedale Maggiore Policlinico, 20122 Milan, Italy; 3 Fondazione IRCCS Galeazzi, 20157 Milano, Italy; hijmatgar@gmail.com; 4 School of Dentistry, Royal College of Surgeons in Ireland, D02 YN77 Dublin, Ireland; 5 Department of Diagnosis and Surgery, Araçatuba Dental School of Sao Paulo, Sao Paulo 16015-050, Brazil; francisley.avila@unesp.br

**Keywords:** dental implants, bone augmentation, GBR, collagen membrane

## Abstract

*Background and Objectives*: Adequate buccal bone thickness is critical for long-term peri-implant health and stability. When residual alveolar bone volume is insufficient, guided bone regeneration (GBR) is a widely adopted technique. While non-resorbable membranes provide structural support, they carry a higher risk of complications and require secondary surgery. Resorbable collagen membranes, offer promising biological properties and easier clinical handling, yet clinical data remain limited. This prospective cohort study aimed to evaluate the clinical and radiographic outcomes of horizontal GBR using a native, non–cross-linked resorbable porcine pericardium membrane fixed with titanium pins, in conjunction with simultaneous implant placement. *Materials and Methods*: Eighteen patients (26 implants) with horizontal alveolar defects (<6 mm) underwent implant placement and GBR with deproteinized bovine bone mineral and a porcine pericardium collagen membrane. Horizontal bone gain and buccal bone thickness were measured at baseline and 6 months post-operatively. Post-operative complications, patient-reported outcomes (PROMs), and peri-implant tissue health were assessed up to 1 year post-loading. *Results*: Mean bone gain was 2.95 ± 0.95 mm, and all sites achieved a buccal bone thickness ≥ 1.5 mm. No membrane-related complications occurred. PROMs revealed low morbidity. At 1-year follow-up, marginal bone loss averaged 0.54 ± 0.7 mm, mean probing depth was 2.79 ± 0.78 mm, 92% of sites exhibited keratinized mucosa ≥ 2 mm. *Conclusions*: Native resorbable porcine pericardium membranes, when combined with DBBM and mechanical stabilization, seem to be effective for horizontal bone regeneration.

## 1. Introduction

Bone augmentation procedures simultaneous to implant insertion are commonly performed to improve the peri-implant hard tissue support. This is strictly related to the peri-implant phenotype characteristics of healthy implants, which include the peri-implant bone thickness [1]. Accordingly, the buccal bone thickness (BBT), defined as the horizontal dimension of the bone that supports an osseointegrated implant, can be thin (<2 mm) or thick (≥2 mm). It has been claimed that a minimum BBT of at least 2 mm is recommended to ensure long-term success [2,3]. Indeed, a thick BBT has been associated with reduced peri-implant bone loss and improved stability of soft tissues, while thinner buccal walls are more prone to resorption, potentially compromising long-term outcomes [2]. Similarly, it has been stated that implants showing a BBT of <1.5 mm after placement are more likely to show increased vertical bone loss and less favourable clinical and radiographic outcomes [4]. It appears therefore clear that a minimum amount of BBT for complete coverage of exposed implant surfaces is essential to ensure long-term success of implant restorations, as well as an adequate aesthetic outcome and a proper biomechanical support of the prosthesis. However, in many cases following implantation, the residual bone volume at the implant site is insufficient, necessitating augmentation procedures.

In such circumstances, guided bone regeneration (GBR) applied in both simultaneous and delayed approaches, has become a well-established technique [5,6]. The GBR therapeutic protocol involves surgical placement of a cell-occlusive membrane facing the bone surface, in order to physically seal off the bone defect in need for regeneration from the ingrowth of soft tissue cells. Furthermore, the membrane creates and maintains a secluded space, thus providing an ideal environment for recruitment and proliferation of osteoprogenitor cells, differentiation along the osteoblastic lineage and expression of osteogenic activity [7]. Interestingly, besides the passive mechanical role of barrier membranes, experimental evidence also suggests an active role of the membrane compartment per se in promoting the regenerative processes in the underlying defect during GBR [8]. In this context, various non-resorbable and resorbable membrane materials have been used in experimental and clinical studies. In synthesis, the main limitation of non-resorbable barriers is the need for an additional surgery for membrane retrieval. Furthermore, a frequent complication associated with non-resorbable membranes is soft tissue dehiscence and membrane exposure; this may be followed by infection, which can jeopardize the outcome of the bone regeneration [9,10,11]. By contrast, bioabsorbable membranes offer many advantages compared with non-resorbable barriers. Apart from the fact that there is no need for a second surgical intervention to remove the membrane, they present improved soft tissue healing, good incorporation in the host tissues and rapid resorption in case of exposure, thus preventing the bacterial contamination of the defect. Such degradable membranes in the form of collagen type I and III have been tested in animal studies and found to be effective in bone regeneration in humans [12,13,14,15,16].

The most common resorbable natural polymer membranes are collagen membranes (CMs). Besides the said advantages, collagen is also known for its low immunogenicity, haemostatic properties, and its ability to stimulate rapid vascularization [17]. In contrast, the main disadvantages of collagen-based membranes are their lack of rigidity and fast degradation kinetics by endogenous collagenases, so that the barrier function may not remain long enough for tissue regeneration [18]. Different methods have been used to improve the mechanical properties in terms of collagen matrix stability in order to slow down their degradation rate. To this end, cross-linked collagen membranes (CCM) have been developed to prolong the resorption rate by changing fibril orientation, thickness, or pore size [19]. However, such processes have been shown to impair bone-forming cell response and tissue integration [17]. Therefore, improvements of collagen structure and thickness modifying collagen sources, extraction methods and manufacturing processes have been conducted in order to overcome such drawbacks [20].

From a mechanical standpoint, a factor that can influence the quantity and shape of the newly formed bone is the stability of the blood clot populating the graft material. This is more evident at the coronal region of the bone defect, where the tension caused by sutures and muscle activity is higher. These centripetal forces can lead to a collapse of the membrane toward the defect, resulting in a shrinkage of the volume occupied by the graft. In this context, the use of titanium pins may be effective to stabilize the membrane, improve its tension and minimize micro-movements, ultimately reducing graft contraction [21,22,23,24,25].

As recently underlined, although regenerative procedures have been applied for several decades, there are still knowledge gaps and uncertainties in relation to some factors that can impact on the expected GBR outcomes, including barrier properties and clinical handling [26]. In view of the above, the aim of this study was to evaluate the clinical and radiographic outcomes of GBR procedures using a native, non–cross-linked resorbable CM fixed with titanium pins in case of simultaneous implant placement, from both surgical- and patient-centred outcomes.

## 2. Materials and Methods

### 2.1. Study Design

The research protocol of the present investigation has been approved as a monocentric prospective cohort study by the local Institutional Review Board (ID #448_2016bis).

The trial was registered in ISRCTN with the identifier ISRCTN17399060.

All patients have been treated by the same team at the Implant Centre for Edentulism and Jawbone Atrophies, Maxillofacial Surgery and Odontostomatology Unit, Fondazione IRCCS Cà Granda Maggiore Policlinico Hospital, Milan, Italy. Patient were enrolled between June 2017 and June 2022. All patients received thorough explanations and signed a written informed consent form prior enrolment. All procedures were conducted according to the principles outlined by the World Medical Association Declaration of Helsinki as a statement of ethical principles for medical research involving human participants. each participant received detailed information and gave written informed consent.

### 2.2. Study Population

Patients were recruited according to specific inclusion and exclusion criteria. The former included (1) male and non-pregnant/lactating female patients aged 18 years or older; (2) presence of mono-edentulism or partial edentulism up to two adjacent teeth located in the upper or in the lower jaw; (3) residual horizontal ridge thickness < 6 mm requiring bone augmentation [6]; (4) primary stability of the placed implant; (5) loss/extraction of teeth occurred at least 2 month before the date of the supposed GBR, with healed soft tissues at the implant site; (6) American Society of Anaesthesiologists (ASA) physical status classification 1 or 2. Patients were excluded if presenting with full mouth plaque and bleeding scores ≥ 25%, smoking habits (>10 cigarettes/day), uncontrolled diabetes, and in therapy with antiresorptive agents.

### 2.3. Implant Placement and GBR (T0)

**Figure 1:**
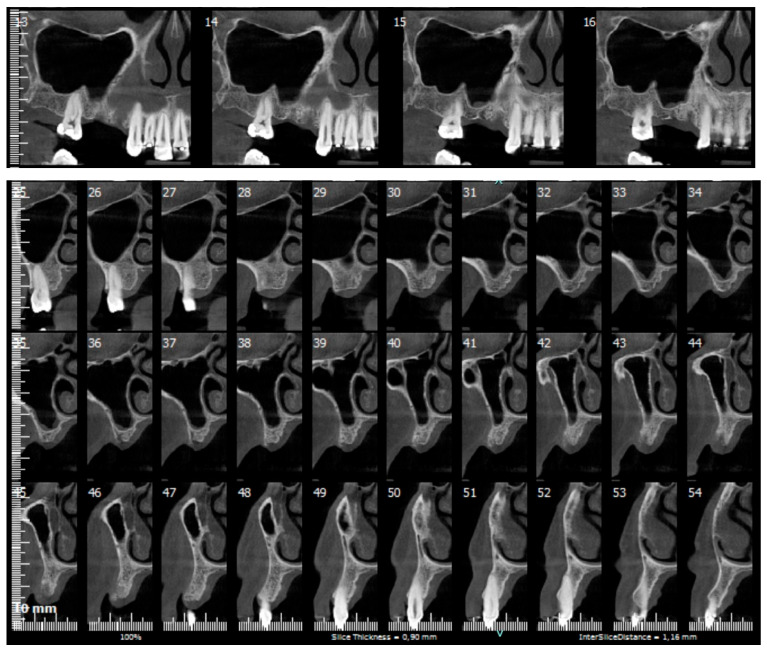
Preoperative cone-beam computed tomography.

Before the surgical procedure, hydrocolloid impressions were taken and poured with dental stone. A surgical guide implemented with a diagnostic wax-up of the crown to be replaced was manufactured with a ~2 mm-diameter guiding hole to achieve a correct prosthetically driven implant placement. A cone-beam computed tomography (CBCT) scan was acquired to evaluate the edentulous site and verify the feasibility of an implant placement with simultaneous GBR according to the prosthetic waxing (Figure 1).

Presurgical preparation consisted of chlorhexidine digluconate 0.2% mouthwashes (Corsodyl, GlaxoSmithKline, Brentford, UK) for 2 min and an extraoral scrub with povidone–iodine solution (Betadine, Viatris, Milano, Italy). Intramuscular injections of 4 mg/mL of dexamethasone sodium phosphate (Soldesam, Laboratorio Farmacologico Milanese, Varese, Italy) were performed to reduce postoperative edema.

**Figure 2:**
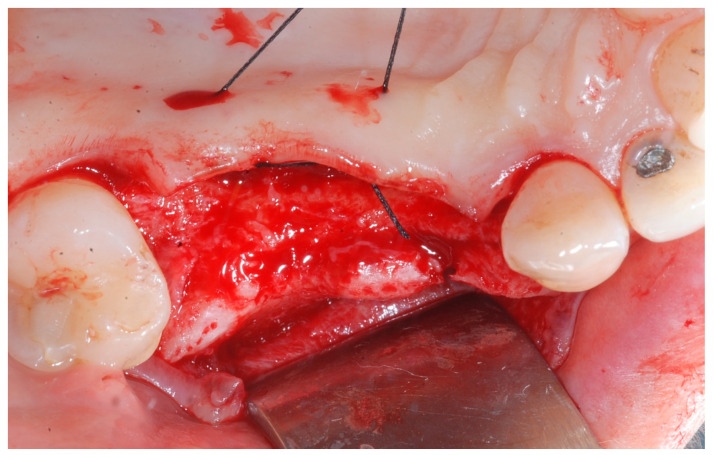
Trapezoidal flap elevation and initial bone thickness.

Local anesthesia was obtained with mepivacaine 20 mg/mL with epinephrine 1:100.000 (Optocain, Molteni Dental, Milan, Italy) injections. To raise a mucoperiosteal flap, a crestal incision followed by oblique releasing incisions were made to allow for a wide flap basis as well as sufficient access to the defective ridge area. The flaps were carefully raised using tissue elevators. The bone ridge was examined and any soft tissues remaining on the crest were meticulously removed with a surgical curette (Figure 2).

**Figure 3:**
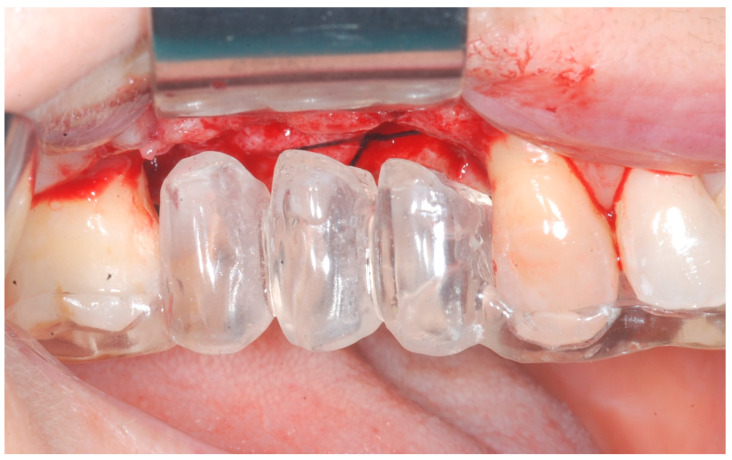
Surgical stent used for implant positioning.

**Figure 4:**
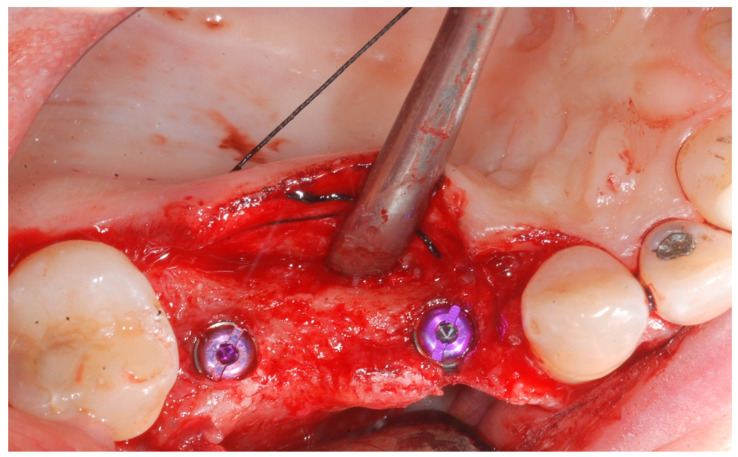
Implant positioning and cover screws in place.

At this point, implants were placed according to the manufacturer’s instructions in a prosthetically ideal position with the aid of the surgical stent (Figure 3 and Figure 4).

**Figure 5:**
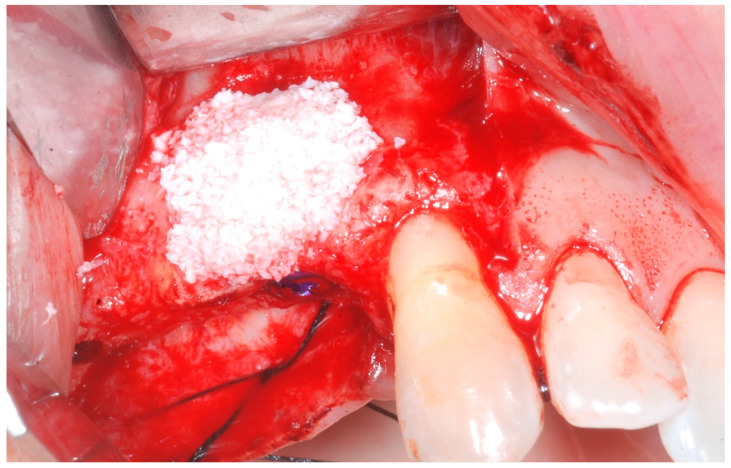
DBBM graft to cover the defect area.

**Figure 6:**
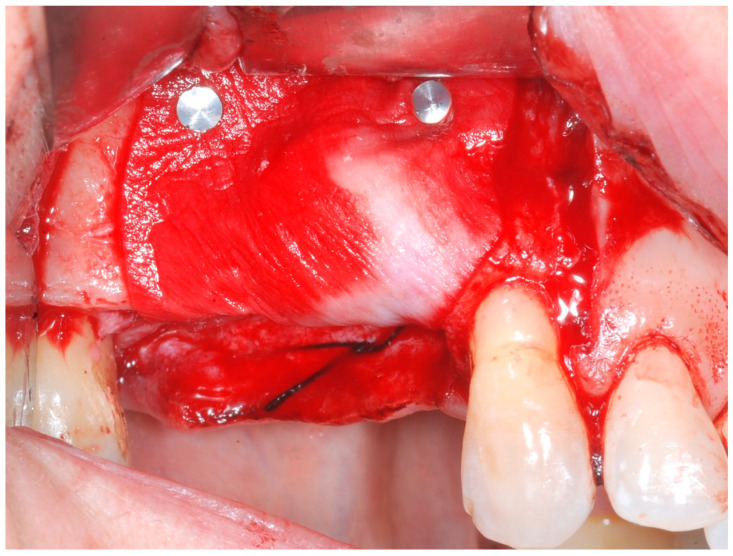
CM fixated with titanium pins to cover the graft.

The cortical bone plate was perforated at numerous locations using a round bur in order to allow access of the cells from the bone and bone marrow to the area of regeneration. Subsequently, granules of cancellous deproteinized bovine bone mineral (DBBM) (Bio-Oss, Geistlich AG, Wolhusen, Switzerland), were placed in the defect area (Figure 5). A collagenous resorbable membrane (T-Gen, HYUNDAI BIOLAND Co., Ltd., 162, Gwahaksaneop 3-ro, Ochang-eup, Cheongwon-gu, Cheongju-si, Chungcheongbuk-do, 28125, Republic of Korea) was shaped and trimmed to cover the graft and to extend 2–3 mm onto the intact bony borders of the defect. The membrane was hydrated and the fixation was accomplished using fixation pins (MC Bio S.r.l., Como, Italy) (Figure 6).

**Figure 7:**
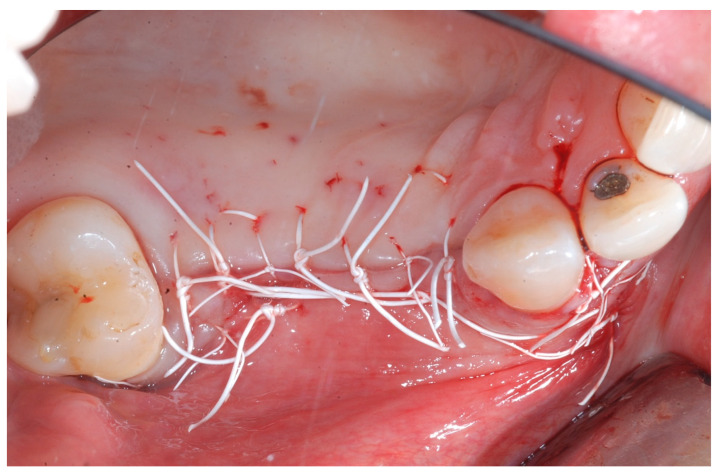
Tension-free sutures.

**Figure 8:**
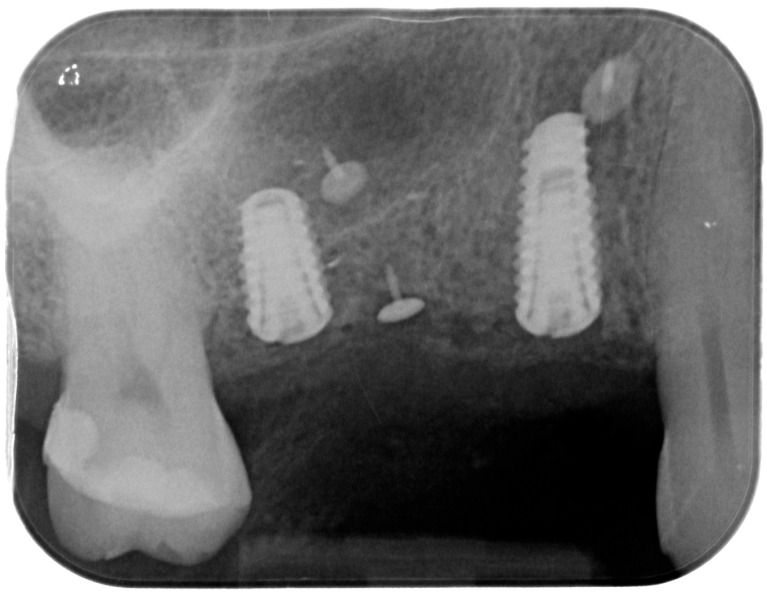
Postoperative intraoral X-ray.

Releasing incisions were made through the periosteum at the base of the flap in order to allow tension-free adaptation of the wound margins. Horizontal mattress sutures as well as single interrupted sutures (CV-5 and CV-7, Gore-Tex; W.L. Gore & Associates, Flagstaff, AZ, USA) were placed to achieve healing by primary intention (Figure 7 and Figure 8).

All patients underwent antibiotic regimen with amoxicillin clavulanate 1 g (Augmentin, GlaxoSmithKline, Brentford, UK) twice daily starting 1 day before surgery and then twice daily for 6 days. After the surgery, an anti-inflammatory agent (Ketoprofen, Orudis, Aventis Pharma, Origgio, Varese, Italy) was prescribed. Patients were also instructed to rinse twice daily with a 0.2% chlorhexidine solution and to refrain from mechanical plaque removal in the surgical area for 2 weeks. Two weeks following augmentation surgery, sutures were removed. Follow-up visits were scheduled every 6–8 weeks until re-entry surgery.

### 2.4. Re-Entry Surgery (T1)

**Figure 9:**
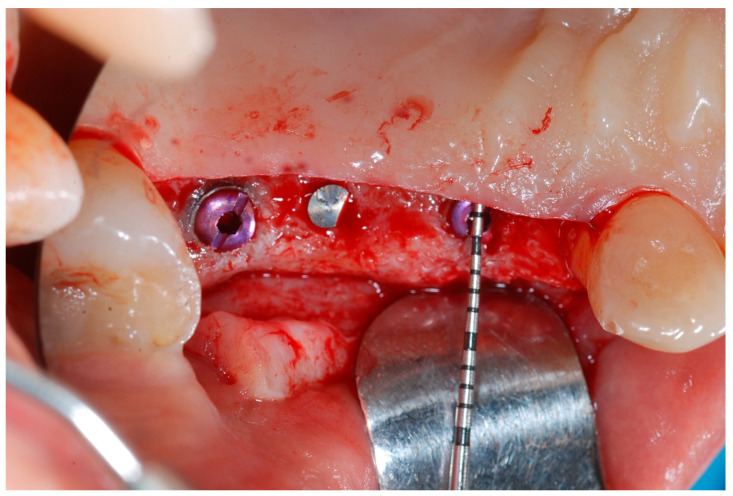
Reopening surgery showing no membrane residues and obtained buccal bone thickness.

**Figure 10:**
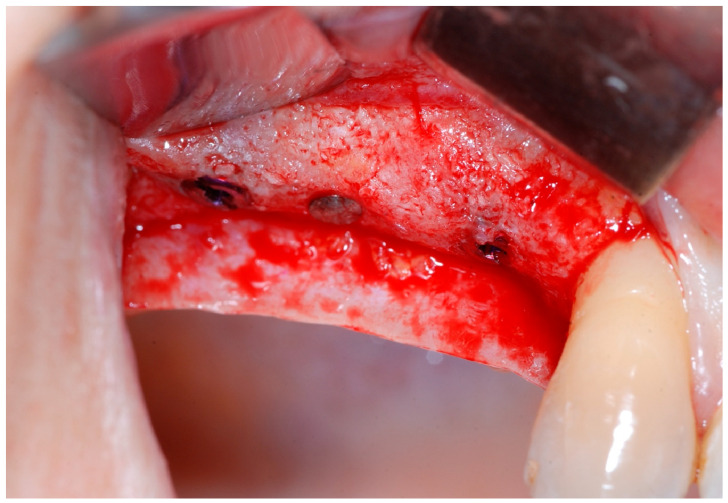
Reopening surgery showing no membrane residues and obtained buccal bone thickness.

Six months following augmentation, re-entry surgery was carried out to uncover the implants and connect the healing abutments. Following chlorhexidine rinses and the injection of local anesthetics, crestal incisions as well as releasing incisions along the same lines as the ones during augmentation surgery were performed. Mucoperiosteal flaps were raised in order to visualize the augmented bone volume (Figure 9 and Figure 10).

**Figure 11:**
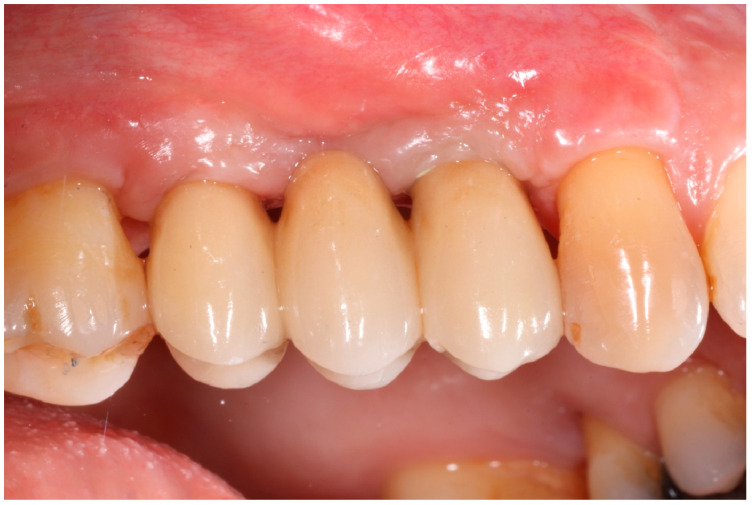
Delivery of definitive prosthesis.

**Figure 12:**
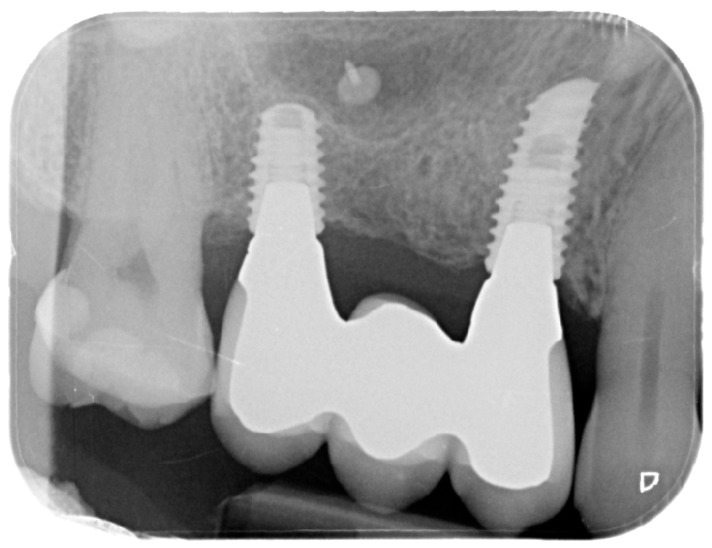
Intraoral X-ray at delivery of the final prosthesis.

**Figure 13:**
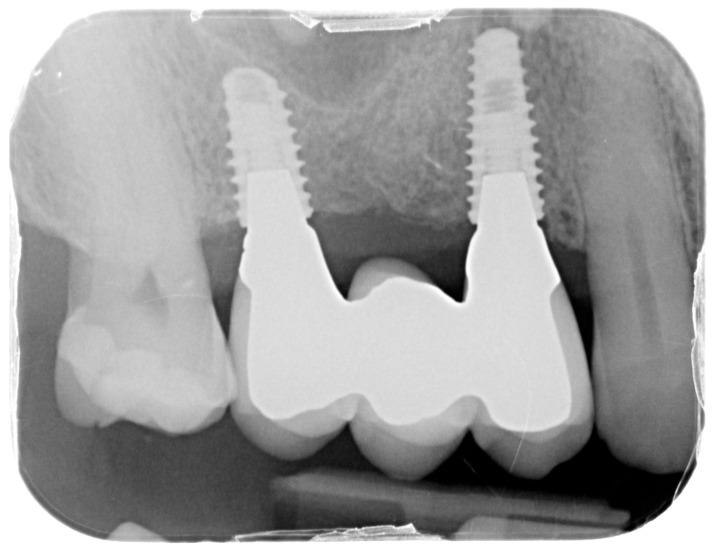
Intraoral X-ray at one year follow-up.

Healing abutments were screwed to the implants and the flaps were sutured. Additional peri-implant plastic surgery procedures (connective tissue grafts or free gingival grafts) were performed when needed in order to have at least 2 mm of attached keratinized mucosa. The prosthetic phases began upon healing of the soft tissues, and definitive screw-retained prostheses were finally delivered (Figure 11, Figure 12 and Figure 13).

### 2.5. Study Endpoints

The evaluation of the horizontal bone gain (hBG), obtained following the GBR procedure, was the primary endpoint. This was assessed clinically by means of two intra-operative measurements: during the GBR surgery (T0) and subsequently at the implant uncovering (T1) after 6 months of healing. The measurements of the entire buccal-lingual/palatal ridge thickness was performed with a Castroviejo surgical calliper 2 mm apically from the top of the crest and rounded to the nearest mm at the centre of the implant site. The remaining teeth adjacent to the defect and the surgical guide were used as reference points, in order to repeat the exact mesio-distal measurement position during the second examinations at T1. Furthermore, at T1 the BBT was measured with a periodontal probe (PCP-UNC 15, Hu-Friedy, Chicago, IL, USA) rounded to the nearest 0.5 mm at the centre of the implant site.

The clinical evaluation of the biocompatibility of the CM was performed throughout the healing period from T0 to T1. During the 6-month healing period, the complication rate was assessed to evaluate the biocompatibility of the CM in relation to the post-operative complications, namely flap dehiscences, membrane exposures, presence of infections and fistulae with pus discharge amongst others. At T1, the macroscopic appearance of the reconstructed site was assessed intra-surgically to evaluate graft integration and detect any remnants of the CM or its complete resorption due to the degradation phases.

With respect to patient-reported outcome measures (PROMs), the evaluation of the post-operative morbidity was carried out by means of a specific form. Indeed, a questionnaire about the postoperative course from 5 to 6 h postoperatively (before intake of the prescribed analgesics) and until the third postoperative day was filled by the patients. A 100 mm visual analogue scales (VAS) with extreme end-points was used to record the following: the intensity of postoperative pain on the day of surgery, 1, 2 and 3 days after surgery (no and extreme pain), the severity of swelling on the day of surgery, 1, 2 and 3 days after surgery (no and severe swelling), and the severity of bleeding from the wound on the day of surgery, 1, 2 and 3 days after surgery (no and severe bleeding). Furthermore, on the day of the surgery, the patients scored their satisfaction with their perception of the operation (not and very unpleasant). The questionnaires were collected at the time of the first post-operative recall 7 days after T0. By means of a ruler, the VAS scores were measured and rounded off to the nearest integer.

For the evaluation of the mesial and distal MBL, intraoral digital radiographs were taken using the long-cone paralleling technique with the central beam directed to the alveolar crest. Periapical radiographs were taken at the delivery of the prosthesis and after 1 year of prosthetic loading (T2). The mesial and distal MBL, i.e., the distance between the top of the implant shoulder and the first visible bone-to-implant contact, were measured at the mesial and distal aspect with a 10–15× magnification using an image analysis programme (ImageJ v 1.49, NIH, Bethesda, MA, USA). The length of the implant was used as known measure for the calibration and determination of the exact magnification and distortion of the images. All measurements were performed by two examiners to the nearest 0.1 mm. In case of disagreement, the evaluation was re-done and results discussed until an agreement was found.

To evaluate soft tissue health, peri-implant probing pocket depth (PPD), keratinized mucosa width (KMW), and bleeding on probing (BoP) were registered at T2 with a periodontal probe (PCP-UNC 15, Hu-Friedy, Chicago, IL, USA). PPD was measured at six sites around each implant; KMW defined as the apico-coronal distance from the mucogingival junction to the gingival margin was measured at the zenith of each prosthetic crown; BoP was recorded as present or absent at each site.

### 2.6. Statistical Analysis

The hBG was used as the primary endpoint to calculate the sample size by means of dedicated software (G*Power 3.1, Heinrich-Heine University, Dusseldorf, Germany). Assuming an expected mean hBG of 4 mm, a standard deviation of 3 mm, a pre- and post-intervention correlation of 0.7, a two-tailed alpha of 0.05, and a power of 80%, 10 subjects were required. Considering the event of some dropouts and the possibility to increase the power of the study, it was decided to extend the initial study population at 20 patients. A descriptive statistic was performed to demonstrate the distribution of data, frequencies, percentages, mean, standard deviation (SD), standard error (SE), 95% confidence intervals. Inferential tests included *t*-tests to compare MBL and PPD between groups with different BBT, chi-square tests to examine categorical relationships (e.g., BoP and KMW), and regression analysis to explore potential predictors of PPD.

## 3. Results

**Figure 14:**
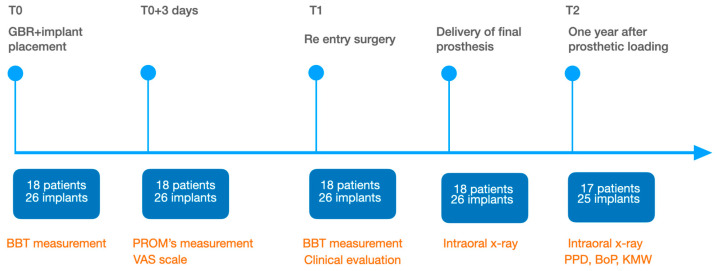
Timeline of performed measurements.

Overall, 20 subjects were initially scheduled. Two of them refused to undergo bone augmentation procedures and opted for a removable prosthesis. A total of 18 participants with 26 implants examined were included in this study, with 21 (80.77%) being female and 5 (19.23%) male. The mean age of the participants was 62.96 ± 12.30 years, ranging from 32 to 78 years at the time of the surgical phase. Implant diameters ranged from 3.5 to 4 mm. During the study period, one dropout was recorded after delivery of the prosthesis. This patient moved to another city and was not available for the follow-up examinations, therefore only data up to T1 were taken into considerations. In the end, 25 surgical sites were evaluated (Figure 14).

**Figure 15:**
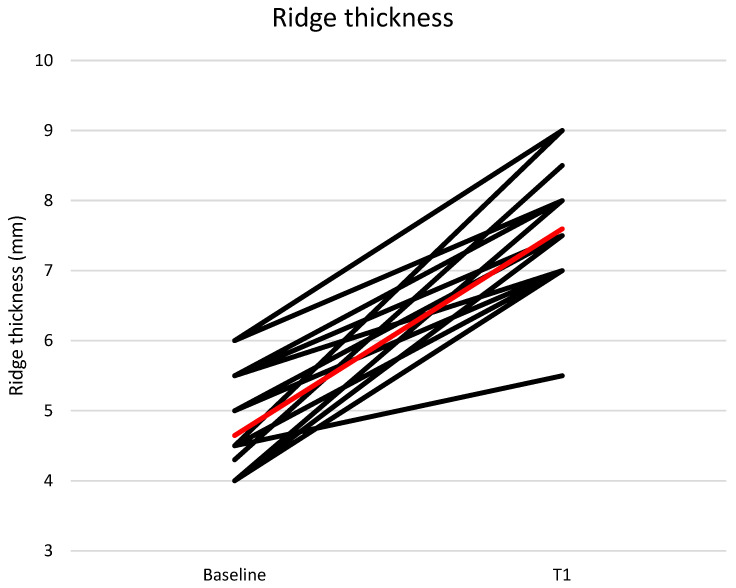
Ridge thickness at baseline and T1. Mean values are represented by the red line.

The mean buccal-lingual/palatal ridge thickness at the implant site was 4.65 ± 0.65 mm at T0 and 7.60 ± 0.81 mm at T1 (Figure 15).

**Figure 16:**
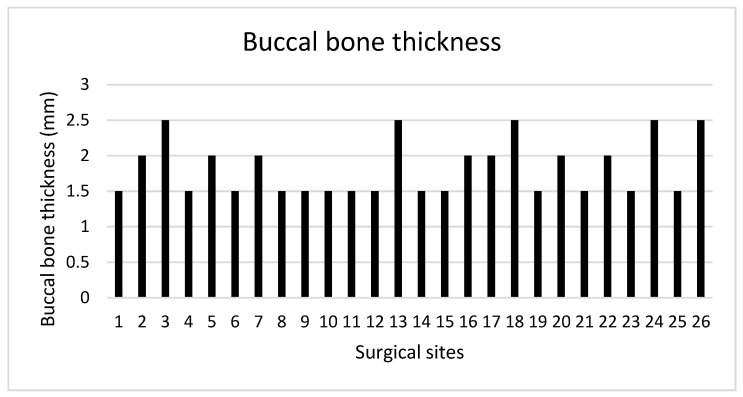
Buccal bone thickness at T1.

The mean hBG achieved following implant placement and simultaneous GBR was 2.95 ± 0.95 mm. In all surgical sites, the BBT measured at T1 at each implant site was ≥1.5 mm, with a mean value of 1.83 ± 0.39 mm (Figure 16).

A total of 13 surgical sites were characterized by a BBT < 2 mm, whereas the remaining 12 showed a BBT ≥ 2 mm.

During the 6-month period from T0 to T1, healing proceeded uneventfully in all patients, with no major complications or biocompatibility issues. No allergic reactions were observed. No complications such as flap dehiscence, membrane exposure, wound infection, fistulae with pus discharge, or foreign body reactions were noticed. None of the surgical sites had to be re-opened earlier. During the re-entry phase, in each surgical site no membrane remnants could be detected. Augmented newly formed hard tissue was observed clinically, with macroscopic features of healthy, bleeding, newly formed bone. Remnants of DBBM granules were occasionally visible and appeared firmly attached to the regenerated hard tissue, with no macroscopic signs of fibrous encapsulation. A thin layer of pseudo-periosteum with a thickness < 1 mm was seldomly observed. In all cases, implants appeared clinically stable, with no residual bony dehiscences or fenestrations at the buccal aspect.

**Figure 17:**
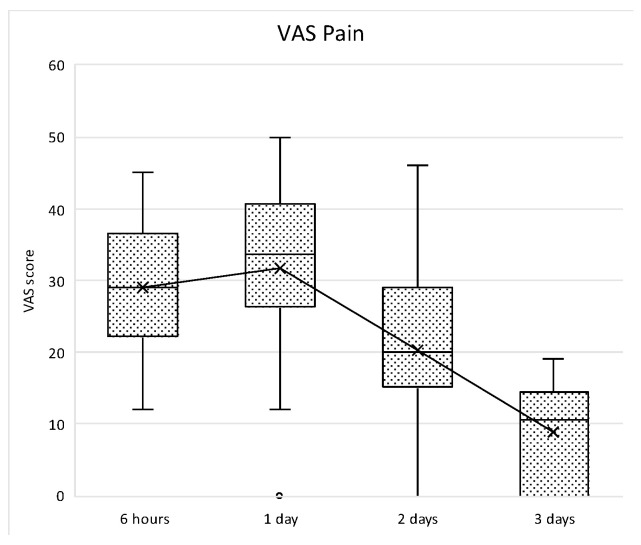
Box plot showing VAS scores related to subjective pain perception amongst each study intervals. “x” symbol represents mean values at each timepoint.

With respect to PROMs, mean values for pain indicate that pain perception normally increased within the first post-operative day, and then decreased thereafter (Figure 17).

**Figure 18:**
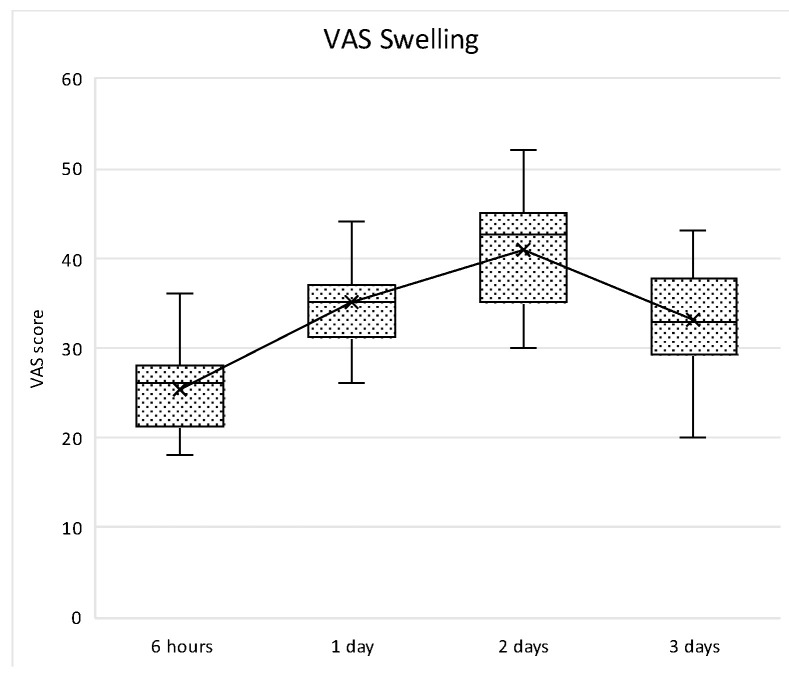
Box plot showing VAS scores related to subjective swelling perception amongst each study intervals. “x” symbol represents mean values at each timepoint.

The increase was not statistically significantly different between 6 h and 24 h, then a statistically significant decrease was observed between 1 and 2 days (*p* = 0.006) and between 2 and 3 days (*p* = 0.006). Similarly, swelling perception normally increased within the first two post-operative days with a peak at 48 h, and then decreases thereafter. In all time points, the differences were statistically significant (*p* < 0.005) (Figure 18).

Box plot of VAS pain scores at different study intervals. The horizontal line inside each box represents the median value, while the box edges indicate the interquartile range (25th–75th percentile). The whiskers represent the minimum and maximum values. The dotted line connects the mean values at each time point to illustrate the overall trend.

**Figure 19:**
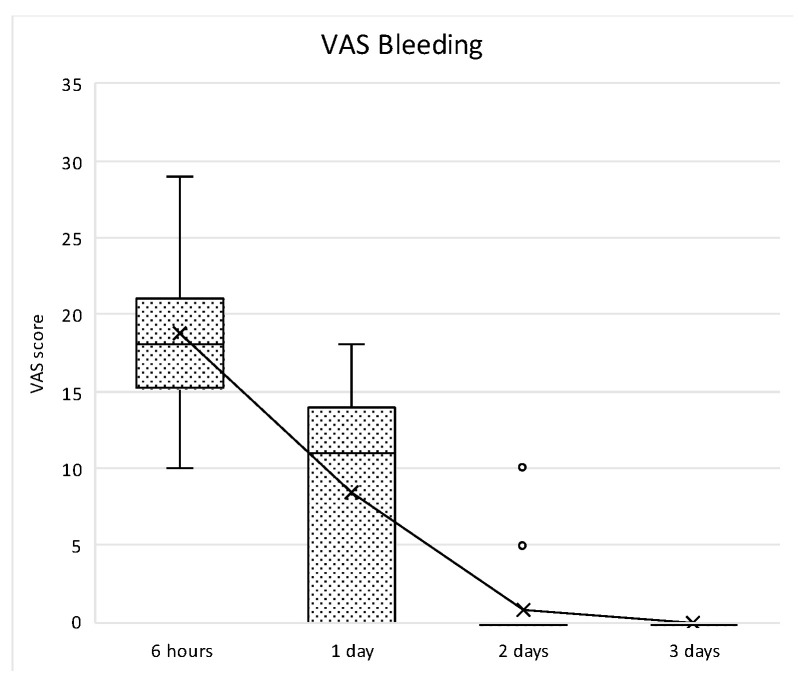
Box plot showing VAS scores related to subjective bleeding perception amongst each study intervals. “x” symbol represents mean values at each timepoint.

Bleeding perception was high during the first 24 h, and then decreased thereafter, reaching no bleeding perception at day 3. After the peak at 6 h, the decrease was statistically significant up to the second post-surgical day (*p* < 0.001), whereas no statistically significant differences were observed between day 2 and 3 (*p* = 0.948) (Figure 19).

Box plot of VAS pain scores at different study intervals. The horizontal line inside each box represents the median value, while the box edges indicate the interquartile range (25th–75th percentile). The whiskers represent the minimum and maximum values. The dotted line connects the mean values at each time point to illustrate the overall trend.

The status of peri-implant hard and soft tissues was assessed at T2, one year from the prosthetic loading. Data of 17 participants and 25 surgical sites were available, as one drop-out was recorded. The patient moved to another city and was not available for the follow-up recall. The overall mean MBL considering both mesial and distal aspects was 0.54 ± 0.7 mm. The mean BoP rate was 38.1%, while the mean PPD was 2.79 ± 0.78 mm with a maximum value of 4.5 mm. There were no statistically significant differences in MBL, PPD, BoP, or KMW based on BBT (*p* > 0.05). The correlation between BBT and clinical outcomes was weak and not statistically significant. Specifically, thicker buccal bone was associated with a slight reduction in PPD (coefficient: −0.33), but this effect was not statistically significant (*p* > 0.05). In contrast, BoP was strongly associated with increased PPD (coefficient: 1.04, *p* < 0.001), indicating that patients with higher BoP scores tended to have deeper PPD. There was no significant effect of KMW on PPD (*p* > 0.05).

## 4. Discussion

Alveolar bone reconstruction by means of GBR harnesses the principle of cell occlusion with a barrier that separates the defect site from soft tissues, preventing rapid invasion by non-osteogenic cells and allowing undisturbed bone healing. In the present study, the efficacy of a native, non–cross-linked resorbable CM fixed with titanium pins has been evaluated during horizontal GBR procedures. Key clinical and radiological parameters including hBG, MBL, PPD, BoP, and KMW were assessed alongside self-reported PROMs.

The results showed an overall mean hBG of approximately 3 mm, with all implant sites characterized by a BBT ≥ 1.5 mm. This confirmed that the native, non–cross-linked CM combined with DBBM and titanium pin fixation was able to provide the three-dimensional stability necessary to maintain the graft space and prevent soft tissue ingrowth and collapse, thereby supporting new bone formation. Accordingly, a recently revised decision tree on horizontal ridge augmentation suggested the use of GBR with resorbable CM and bone graft simultaneously to implant placement when the desired hBG is <3 mm [27]. In such cases, the use of fixation devices has been advocated to reduce displacement of the bone substitute resulting in a partial collapse of the CM in the coronal portion of the augmented site following wound closure and soft tissue pressure [21]. In this respect, native CMs stabilized with bone pins yielded higher total augmented area and augmented tissue thickness compared to non-stabilized native CMs, while no differences were noted with cross-linked CMs [28].

Notably, the assessment of different BBT comparing thin (<2 mm) versus thick (≥2 mm) biotypes revealed no significant differences in MBL, PPD, BoP, or KMW at T2. These observations suggest that, in our cohort, the use of a native non–cross-linked collagen membrane in combination with DBBM and rigid fixation may help maintain clinical outcomes in sites with thin biotypes, which are generally considered at higher risk for peri-implant bone loss and soft tissue recession [2,29,30]. In thin biotype sites, concerns typically involve reduced vascularization, limited soft tissue thickness, and increased susceptibility to inflammatory insult, potentially compromising hard and soft tissue stability. Our data indicate that the regenerative approach used allowed all implant sites to achieve a BBT ≥ 1.5 mm. However, it should be noted that these findings are based on a single-cohort study without a comparative group of non-pinned membranes, and further research is needed to confirm the potential benefits of titanium pin fixation in thin biotypes. Not only an adequate BBT has been recommended to promote long-term peri-implant health, but also a bone width of 7 mm for a 4 mm diameter implant, which is in line with the data reported herein at T1 [4]. These assumptions are supported by pre-clinical models showing that on average, a baseline BBT < 1.5 mm is exposed to approximately 4 mm of vertical bone loss under spontaneous healing, while in scenarios where BBT is ≥1.5 mm, vertical bone loss is limited to about 0.1 mm. At the same time, greater PPD, mucosal recession, sulcular bleeding index and suppuration were noted under a baseline BBT < 1.5 mm when compared to BBT ≥ 1.5 mm [Monje 2019 3]. Although it is difficult to identify a specific BBT threshold that guarantees stability of the residual alveolar bone in the buccal wall after implant placement, pre-clinical and clinical evidence indicates that BBT < 1.5–2 mm tended to show greater vertical bone loss, mucosal recession and BBT reduction over time [2].

In the present study, bone augmentation was obtained with a natural collagen porcine pericardium membrane. Histological and clinical evidence supports that although cross-linked CMs offer prolonged resorption time, they may impede tissue integration, provoke foreign-body reactions and show high exposure rates [31]. Considering the latter, cross-linked CMs has shown residues of the cross-linked substance potentially leading to cytotoxic issues [32]. In contrast, resorption pattern of native CMs allows maintaining their inherent biological properties, fostering cell attachment, vascular ingrowth, and a balanced inflammatory response. In this matter, survival and adhesion of gingival fibroblasts on cross-linked CMs seem to be less than natural CM [32]. Interestingly, non-cross-linked membranes are also more affordable for premature transmembranous formation of blood vessels than cross-linked membranes, hence improving early blood supply of the soft tissues thus reducing the risk of exposure [16]. In favour of native porcine pericardium membranes, considerable biocompatibility in terms of cell proliferation and adhesion and potential osteoconductive and osteoinductive properties have also been highlighted both in vitro and in vivo [27]. Similarly, a comparison of porcine pericardium, bovine tendon, and cross-linked bovine tendon confirmed that the native CMs can provide a better surface for soft tissue integration in terms of cell adhesion and proliferation [31]. All taken together, it has been speculated that resorption time may not be the primary concern for GBR, as the membrane is only necessary during the initial week of healing [33]. In the present study, no complications were observed between T0 and T1, strengthening the biocompatibility of natural collagen porcine pericardium membranes during GBR procedures. Furthermore, complete membrane resorption was consistently observed at re-entry, with no residual membrane material detected, underscoring the predictable degradation profile and favourable tissue interaction of the native collagen barrier.

These favourable results had an impact on the PROMs that further underscored the technique’s acceptability. Pain and swelling peaked within the first 24–48 h and then declined significantly. Bleeding perception was high during the first 24 h but decreased rapidly, reaching negligible levels by day 3. In general, the absence of a secondary surgery for membrane removal is an additional known advantage over non-resorbable barriers leading to low patient morbidity and enhanced comfort.

The KMW also did not differ significantly between thin or thick BBT, nor did it correlate with marginal bone levels or implant survival. While the role of KM in peri-implant health remains debated, evidence suggests that adequate width of KM may facilitate oral hygiene and patient comfort without necessarily impacting hard tissue parameters [34]. Moreover, an adequate width of KM around dental implants, ≥2 mm, has been associated with reduced plaque accumulation, tissue inflammation, and bone loss [35]. Contrarily, narrower KMW correlated with increased mucosal recession, higher gingival and plaque indices scores and greater marginal bone loss [36]. In the present cohort, preservation of existing KM during flap design and suturing likely contributed to stable peri-implant soft tissue outcomes, irrespective of baseline KM width. Overall, following adequate soft tissues management, 23 out of 25 sites presented with >2 mm of KMW at T2. This reinforces the concept that, when using biocompatible regenerative materials and meticulous surgical techniques, the presence of a minimal band of at least 2 mm of KMW is enough to maintain peri-implant health. It should be mentioned that in only 2 out of 25 sites the KMW was <2 mm, making the comparison between thin and thick biotypes difficult if not irrelevant. Furthermore, all patients were enrolled in a strict oral hygiene maintenance programme, which might have played an additional role in the stability of peri-implant tissues at T2.

Overall, these findings suggest that native resorbable CMs, when used in conjunction with DBBM and rigid fixation, constitute an effective and patient-friendly option for horizontal GBR. They combine predictable degradation, favourable tissue integration, and avoidance of a second surgical intervention, without the biocompatibility issues or foreign-body reactions reported for cross-linked devices. Despite the limited sample size and relatively short follow-up period, which may restrict detection of subtle long-term differences, the present results support the broad applicability of native CMs in clinical practice for reliable bone augmentation and implant site development. Future randomized trials with larger cohorts and extended monitoring are warranted to confirm these outcomes across diverse defect anatomies and patient biotypes.

## 5. Conclusions

The results of the present study suggest that natural collagen porcine pericardium membranes in combination with DBBM particles and fixation pins may be effective in horizontal GBR procedures. In all cases, BBT was ≥1.5 mm, with no complications occurred during the 6-month healing period. The role of BBT in preserving healthy hard and soft tissues needs further investigation as MBL, PPD, BoP, and KMW were similar in patients with values below and above 2 mm.

## Data Availability

The raw data supporting the conclusions of this article will be made available by the authors on request.

## Abbreviations

The following abbreviations are used in this manuscript:

BBT	Buccal bone thickness
GBR	Guided bone regeneration
CM	Collagen membrane
DBBM	Deprotenized bovine bone mineral
CCM	Cross-linked collagen membrane
CBCT	Cone beam computerized tomography
hBG	Horizontal bone gain
PROMs	Patient-reported outcome measures
MBL	Marginal bone loss
PPD	Probing pocket depth
BoP	Bleeding on probing
KMW	Keratinized mucosa width
